# Clinical Trial Subjects: “Panning Gold”

**DOI:** 10.3389/fpubh.2013.00053

**Published:** 2013-11-20

**Authors:** John Franklyn Riefler

**Affiliations:** ^1^Medical and Safety Services, ICON Clinical Research, North Wales, PA, USA

**Keywords:** screening subjects, PGE_2_, anti-infective trials, crevicular fluid, Gram stain

Anti-infective drug development depends to a great extent on enrolling the “correct” subjects in Ph II–III clinical trials – i.e., those who can show a therapeutic benefit from the anti-infective therapy.

For example, while at Lederle Labs, I worked on a new indication for MINOCIN^®^-namely, treatment of moderate-to-severe periodontitis. MINOCIN^®^-reduces the numbers of the putative periodontal pathogens *Actinobacillus actinomycetemcomitans* (Aa), *Prevotella intermedia* (Pi), *Porphyromonas gingivalis* (Pg), *Tannerella forsythia*, and *Treponema denticola* [the latter three organisms constitute red-complex bacteria (RCB)]. However, scaling and root planing (S/P), the gold standard treatment removes approximately 90% of plaque and bacteria. We wanted to show increased benefit in Ph II trials with adjunctive use of microencapsulated minocycline hydrochloride (ARESTIN^®^) plus S/P vs. S/P alone. We needed a marker to identify the “correct” patients to enroll.

We consulted four periodontologists who had experience doing this research. The first advised us to use a “clinical marker” to identify active cases of periodontitis. He recommended including subjects with deep periodontal pockets – i.e., two teeth with at least one 7 mm pocket. The trial produced trends, but no clear benefit. The second recommended enrolling subjects with two sites in the same quadrant with at least 7 mm pocket depth (PD). The trial produced trends, but no clear benefit. The third advised us to use a “microbiological marker.” Each subject had to have one or more of the putative periodontal. In addition, two teeth in the same quadrant had to have at least 7 mm PD. Again, we saw trends, but no clear benefit. We discovered the vast majority of sites (≥80%) were not actively breaking down (the equivalent of a “burned out” volcano). We needed sites that had active tissue breakdown. Fortunately, the fourth expert advised us to enroll subjects with two teeth having one site each with PD of at least 6 mm and with prostaglandin E_2_ (PGE_2_) levels >66.2 ng/mL in gingival crevicular fluid. PGE_2_ mediates inflammation. An earlier study in 12 patients with periodontal disease showed subjects with periodontitis had high PGE_2_ levels (mean = 179.5 pg/μL) while subjects with gingivitis had low PGE_2_ levels (mean = 32.1 pg/μL) (1). We screened 400 subjects to find 50 subjects with high PGE_2_ levels, but these subjects had periodontal pockets like “active” volcanos). The trial lasted 6 months duration and used a formulation of minocycline microspheres containing 1 mg minocycline. Finally, with only 48 patients we showed an advantage [clinically significant probing PD reduction, probing attachment level (AL) gain, and reduction of pathogenic microflora] with an antibiotic + S/P vs. S/P alone (2). Ph II an odyssey lasting several years caused my management to ask “why are you not in Ph III yet?” I replied: because we don’t know if we have a drug. Only after reviewing the data from the fourth Ph II trial, did we believe we had a drug.

The knowledge gained from these four Ph II studies led to two identical pivotal Ph III safety and efficacy trials. A total of 748 patients enrolled in both studies. Patients had to have at least 10 teeth remaining in the function dentition, excluding third molars, and at least four teeth with periodontal PD of 6–9 mm and bleeding on probing (BOP) on all four qualifying teeth. In these two adequate and well controlled studies, ARESTIN^®^ plus scaling and root planning showed a statistically significant improvement in PD reduction when compared to scaling and root planning alone at 9 months. These trials led to the approval of ARESTIN^®^(3).

Similarly, in developing everninomycin, a new class of intravenous antibacterial, we wanted to take advantage of its low MIC_90_ (0.2 μg/mL) for *Streptococcus pneumoniae*. Consequently, my team designed a Ph II community-acquired pneumonia (CAP) trial targeting this organism. We screened subjects and required a quality sputum sample with a Gram stain showing lancet-shaped, Gram-positive diplococci (presumptive evidence of *S. pneumoniae*). Sputum and blood cultures confirmed the diagnosis. The two trials had three overlapping active dose groups and ceftriaxone as comparator. We enrolled patients simultaneously in RSA and Latin America to take advantage of the Austral winter. To enroll 90 subjects in each trial, we screened six subjects to find one with a positive sputum Gram stain, but it paid off. All arms in both trials achieved >90% efficacy. However, development of this drug ended due to safety issues.

A prospective study of the diagnostic utility of sputum Gram stain in pneumonia (4) supports our methodology to identify *S. pneumoniae* CAP cases; sputum Gram stain had 0.82 sensitivity for Pneumococcal pneumonia.

Finding patients with active periodontitis to enroll in our Ph II trials was more challenging than anyone imagined. Neither a clinical marker, nor a microbiological marker alone, or in combination were sufficient. Only a clinical marker (PD) in combination with an inflammatory marker (elevated PGE_2_ levels) gave us the patients who would benefit from adjunctive treatment with ARESTIN^®^. Based on this knowledge, a combination of a clinical marker (PD) and an inflammatory marker (BOP) allowed us to enroll the correct patients in Ph III.

Finding patients with CAP due to *S. pneumoniae* was easier. We were only targeting a bacterium. However, we still had to pan for gold by using a sputum Gram stain to enroll the correct patients.
